# Biomechanical Evaluation of the Phases of the Triple Jump Take-Off in a Top Female Athlete

**DOI:** 10.2478/hukin-2014-0004

**Published:** 2014-04-09

**Authors:** Abeer Eissa

**Affiliations:** 1 Faculty of Physical Education for Girls, Helwan University, Egypt.

**Keywords:** take-off, triple jump, female athletes

## Abstract

The triple jump is one of two track and field events in which the athlete aims to maximize the horizontal distance jumped. This jump is comprised of 3 take-off phases (hop, step, and jump), each playing an important role, as they require the jumper to tolerate extremely high forces of impact and to maintain a high level of horizontal velocity. The purpose of the study was to investigate the biomechanical characteristics of the 3 take-off phases in the triple jump in a top female athlete. The 3 take-off phases of the top national female triple jumper were videotaped and analyzed using 2D motion analysis. Three cameras (DSR-SR 68) were placed on the lateral sides of the 3 take-off points, to record the motions of the 3 take-off phases. Results indicated that maximum loss of the horizontal velocity was in the hop phase (1.13 m/s), while the maximum braking time was in the jump phase (0.05 sec). The maximum pushing time was in the jump phase (0.10 s), while the pushing time was equal in the hop and step phases (0.05 s). In conclusion, the success of the triple jump is the result of the physical and technical qualities of the jumper. The excessive loss in horizontal velocity during the 3 take-off phases is the main factor limiting the performance of the top female athlete.

## Introduction

The triple jump is one of the track and field events, which requires the jumper to repeat the generation of maximal force in order to maintain the horizontal velocity during all phases of the jump.

The triple jump consists of a running approach, 3 take-off phases in which the athlete hops on one foot, lands on the same foot, steps onto the opposite foot, and finally jumps and lands in the sand pit (Miladinov and Bonov, 1988).

Triple jump distance depends on the jumper's ability to apply the basic architectural paths during each of the 3 take-off phases (Yu, 1999). During each take-off phase a change in the movement structure and rhythm occurs, which affects the timing of each concentric and eccentric contraction (Koh and Hay, 1990). Therefore, each take-off phase has its own dynamic requirements during the braking and propulsive phases.

Accordingly, force distribution (magnitude, direction) and time of effect during each take-off phase both play an important role in the triple jump (Hay, 1999). Contact between the foot and the ground leads to a decrease of the vertical and horizontal velocity of the jumper.

Studies of world female triple jumpers have shown that horizontal velocity during the approach phase is between 9.31–9.36 (m/s) (Yu, 1996). Horizontal velocity of the 3 take-off phases, the hop, step and jump, are between 8.4 – 8.86, 7.58 – 8.22, and 6.46 – 7.34 (m/s), respectively. Horizontal velocity loss during the 3 take-off phases, the hop, step and jump, are between 0.69 – 0.95, 0.38 – 0.52, 0.85 – 1.05 (m/s), respectively (Yu, 1996; Helmar, 2009; Ai et al., 2011). Vertical velocity of these 3 take-off phases is between 2.09 – 2.49, 1.24 – 1.76, 2.41 – 2.76 (m/s), respectively (Müller and Brüggemann, 1997; Helmar, 2009; Ai et al., 2011); contact time is between 0.103 – 0.110, 0.133 – 0.150, 0.11 – 0.14 s (Helmar, 2009; Ai et al., 2011); pushing time is 0.059, 0.075, and 0.064 s (Yu, 1996); braking time is 0.079, 0.087, and 0.113 s (Jarmo, 2000); and the take-off angle is between 15.02–16.0°, and 9–12.7° (Helmar, 2009; Ai et al., 2011).

Horizontal velocity is gained during the approach and lost primarily due to ground contact during each of the 3 take-off phases. The loss of velocity can be minimized through the utilization of the proper take-off technique at each phase. Accordingly, the challenge that the triple jumper faces is how to maintain the propulsive force during the repetition of the take-off and landing in particular phases of the triple jump.

Studying and analyzing the performance trajectory of the triple jumper provides the optimal evidence to improve technical performance, since the analysis depends on human movement research. The biomechanical analysis is a very important tool in improving technical performance through critical quantitative information to assess the athlete's performance.

This study aims to bridge the gap between theory and practice in triple jump studies. It presents an important new work in key areas such as training and biomechanics. Moreover, there is a significant gap between the world record in the triple jump and the national record of Egypt. Hence, the purpose of the study was to investigate the biomechanical characteristics of the 3 take-off phases in the triple jump in a top Egyptian female athlete.

## Material and Methods

### Participants

The female subject was a member of the Egyptian national team who won the silver medal in Arabian Athletics Championship, 2011. Both successful and failed trials of the subject were examined. Table 1 shows the physical characteristics and records of the participant.

#### Procedures

Measurements were carried out during the competitive period (the last week before the Arab championship) in the Olympic Center in Cairo.

A lateral view videotaping was carried out with three digital cameras (DCR-SR68) 60 frame/s. Cameras were installed perpendicularly to the runway at a distance of 7 m from the middle, with a visual field 6 m for each and with interfere 2 m. The first camera covered the last stride of the approach and take-offs, the second camera covered the landing area of the hop and take-off area of the step, the last camera covered the landing area of the step and the take-off area of the jump.

The athlete warmed up by performing many trials prior to testing.

Six trials were executed by the subject with a full approach and with 4 minutes as recovery between each trial.

Six trials were recorded and biomechanical analysis was performed for four correct trials using the DARTFISH 4.5 program.

The biomechanical analysis and quantitative assessment allowed to obtain the following variables:

- Triple jump distance
- The length of the last stride before take-off
- The length of the hop, step and jump and the percentage of each to the total jump distance
- Horizontal velocity of the last stride take-off approach
- Horizontal velocity of the 3 take-off phases
- Loss of horizontal velocity during the 3 take-off phases
- Vertical velocity during the 3 take-off phases
- Support time, braking time and propulsion time during the 3 take-off phases

Take-off angles during the 3 take-off phases

## Results

Table 1 shows the physical characteristics and records of the tested subject (Table 1). Table 2 shows that the jumping technique was correct with the percentages of the 3 phases equal to 33.21%, 29.40%, 37.39% for the hop, jump and step, respectively. Their take-off angles were 20.5°, 21.5°, and 23.25°, respectively.

Table 3 shows a continual decrease in horizontal velocity from the hop to the step to the jump and a continual increase in vertical velocity from the hop to the step to the jump. The largest loss in horizontal velocity was in the hop, followed by step, and lastly the jump.

Table 4 shows that the longest contact time occured during the jump and the shortest was in the hop. The longest flight time was during the jump and the shortest contact time was during the step. The maximum value of the duty factor was during the hop and the minimum value of the duty factor was during the jump. Table 5 shows that the longest braking time was achieved during the hop and the shortest during the step. The longest propulsion time was achieved during the jump, while the shortest was achieved during the hop. The maximum support coefficient value was during the jump and the minimum during the hop.

## Discussion

The study showed that there are great variances in the distances in the four jump attempts. In this study, performance was characterized by the jump, which is different from all performance models, which involved either a balanced performance or a prevailing hop performance. The constant improvement and increase in total distance between each trial and the next one may be explained by an insufficient warm up prior to the start of the competition.

The variance in lengths and ratios of distances during the performance phases (hop, step, and jump) from one trial to another was related to change in the influential bio–mechanic indicator values (horizontal velocity loss, vertical velocity, take-off angels, time of braking and pushing) and instability of skillful performance of competition.

Considering the high level of performance of the subject, the amount of horizontal velocity achieved during the approach phase in this research sample was quite modest (8.44 m/s), compared to values for other high level athletes (between 9.31 m/s and 9.36 m/s) (Donley, 1991).

The lower mean values of horizontal velocity during the hop, step and jump (7.30 m/s, 6.5 m/s and 5.75 m/s, respectively) observed, compared to those other high level athletes (between 8.4 m/s – 8.86 m/s, 7.58 m/s – 8.22 m/s, 6.46 m/s – 7.34 m/s, respectively) (Helmar, 2009).

Mean values for horizontal velocity loss during the hop, step, and jump (1.13 m/s, 0.81 m/s, and 0.75 m/s, respectively) were higher in the subject, compared to other high level athletes (between 0.69 m/s – 0.95m/s; 0.38 m/s – 0.52 m/s; and 0.85 m/s – 1.05 m/s, respectively) (Dirk and Zebas, 1988; IA et al., 2011).

Mean contact times for the hop and jump in the research subject (0.11 s, 0.15 s, respectively) were almost equal to mean values of the support time for the hop and jump of other high level athletes (between 0.103 – 0.133 s and 0.133 – 0.15 s, respectively). The step support time in the subject was higher than support time for other high level athletes by 0.14 s (0.11 s and 0.14 s) (Helmar, 2009; Ai et al., 2011).

Mean values for braking time during the hop and the jump were lower in the sample (0.06 s and 0.05 s, respectively), compared to these of other high level athletes (0.079 s and 0.113 s, respectively). The step times were equal in the sample (0.09 s) and high level athletes (0.087 s) (Yu, 1999). This may be due to the fact that the mean horizontal velocity values achieved by the subject (8.44 m/s) were lower than those achieved by other elite athletes (9.31 m/s).

Mean values of the push time for the hop were equal in the research subject and other high level athletes (0.050 s). The push time for the step was lower in the studied subject (0.05 s) than push time for the step in other elite athletes (0.070 s) and the push time for the jump was higher in the research subject (0.10 s) compared to other high level athletes (0.064 s) (Donley, 1991).

We could observe higher mean values of the vertical velocity at the take-off for the hop, step and jump (2.25 m/s, 3.41 m/s, and 3.71 m/s, respectively) in the subject, compared to those of other elite athletes (between 2.09 m/s – 2.49 m/s, 1.24 m/s – 1.76 m/s, and 2.41 m/s – 2.67 m/s, respectively) (Harold, 1997; Helmar, 2009; Ia et al., 2011).

The loss of horizontal velocity can be expressed as a liner function of the gain in the vertical velocity during each support phase (James, 1999).

Furthermore, there was an excessive increase in the mean values of the take-off for both the hop and the step in the studied subject (20.5, 21.5, respectively), compared to other elite athletes (between 15.02 – 16 and 9 – 12.7, respectively) (Helmar, 2009).

The increase in the take-off angles leads to an increase in the braking time, which in turn causes an excessive decrease in the horizontal velocity from one take-off phase to the next (Yu, 1999; Ia et al., 2011).

## Conclusion

The success of the triple jump is the result of the physical and technical qualities of the jumper. The excessive loss in horizontal velocity during the 3 take-off phases is the main factor limiting the performance of the top Egyptian female athlete, in comparison to the performance of the world top female jumper.

## Figures and Tables

**Table 1:** Basic characteristics of the studied athlete

**Name**	Enas Gharieb
**Age [year]**	23
**Body mass [kg]**	58
**Body height [cm]**	169
**Best record [m]**	13.2

**Table 2:** Absolute, relative ratios and angle of take-off phases

**Trials**		**1^st^**	**2^nd^**	**3^rd^**	**4^th^**	**x^−^**	**δn**
**Distance (m)**		12.21	12.42	12.55	13.02	12.55	0.34

**Length (m)**	**lastStride**	2.20	2.00	1.95	1.90	2.01	0.13

	**Hop**	4.05	4.23	3.88	4.50	4.17	0.27
	**Step**	3.67	3.64	3.65	3.78	3.69	0.06
	**Jump**	4.48	4.53	5.02	4.72	4.69	0.24

**Relative (%)**	**Hop**	33.20	34.12	30.92	34.61	33.21	1.64
	**Step**	30.08	29.35	29.08	29.08	29.40	0.47
	**Jump**	36.72	36.53	40.00	36.31	37.39	1.75

**Take-off Angle (º)**	**Hop**	19.00	21.00	22.00	20.00	20.50	1.29
	**Step**	20.00	23.00	22.00	21.00	21.50	1.29
	**Jump**	24.00	23.00	24.00	22.00	23.25	0.96

**Table 3:** Horizontal Velocities, Vertical Velocity and Changes of Horizontal CM Velocities at Take-off for Last Stride Hop, Step and Jump Phases

**Trials**		**1^st^**	**2^nd^**	**3^rd^**	**4^th^**	**x^−^**	**δn**
**Distance (m)**		12.21	12.42	12.55	13.02	12.55	0.34

**Horizontal Velocity (m/s)**	**Last Stride**	8.00	8.50	8.50	8.75	8.44	0.31
	**Hop**	6.50	7.75	7.25	7.75	7.31	0.59
	**Step**	6.25	6.25	6.50	7.00	6.50	0.35
	**Jump**	5.50	5.75	5.75	6.00	5.75	0.20

**Change in Horizontal Velocity (m/s)**	**Hop**	−1.50	−0.75	−1.25	−1.00	−1.13	0.32
	**Step**	−0.25	−1.50	−0.75	−0.75	−0.81	0.52
	**Jump**	−0.75	−0.50	−0.75	−1.00	−0.75	0.20

**Vertical Velocity (m/s)**	**Hop**	2.50	2.50	2.00	2.00	2.25	0.29
	**Step**	3.94	3.33	3.33	3.03	3.41	0.38
	**Jump**	3.33	3.93	3.63	3.93	3.71	0.29

**Table 4:** Contact Times, Flight Times and Duty Factor for Hop, Step and Jump Phases

**Trials**		**1^st^**	**2^nd^**	**3^rd^**	**4^th^**	**x^−^**	**δn**
**Distance (m)**		12.21	12.42	12.55	13.02	12.55	0.34

**Support Time (s)**	**Hop**	0.12	0.11	0.11	0.11	0.11	0.01
	**Step**	0.13	0.13	0.12	0.13	0.13	0.01
	**Jump**	0.15	0.15	0.15	0.15	0.15	0.00

**Flight Time (s)**	**Hop**	0.40	0.44	0.39	0.41	0.41	0.02
	**Step**	0.35	0.37	0.37	0.35	0.36	0.01
	**Jump**	0.68	0.67	0.66	0.62	0.66	0.03

**Duty Factor**	**Hop**	3.30	4.00	3.50	3.72	3.63	0.30
	**Step**	2.60	2.70	3.10	2.60	2.75	0.24
	**Jump**	4.60	4.40	4.40	4.10	4.38	0.21

**Table 5:** Braking Times, Pushing Times and Support Factor for Hop, Step and Jump Phases

**Trials**		**1^st^**	**2^nd^**	**3^rd^**	**4^th^**	**x^−^**	**δn**
**Distance (m)**		12.21	12.42	12.55	13.02	12.55	0.34

**Braking Time (s)**	**Hop**	0.07	0.07	0.07	0.05	0.06	0.01
	**Step**	0.08	0.09	0.09	0.09	0.09	0.00
	**Jump**	0.05	0.05	0.05	0.05	0.05	0.00

**Pushing Time (s)**	**Hop**	0.06	0.05	0.05	0.06	0.05	0.01
	**Step**	0.05	0.05	0.05	0.04	0.05	0.01
	**Jump**	0.10	0.10	0.08	0.10	0.10	0.01

**Support Factor**	**Hop**	0.85	0.77	0.69	1.20	0.88	0.22
	**Step**	1.66	1.80	1.80	2.25	1.88	0.26
	**Jump**	2.00	2.00	1.66	2.00	1.92	0.17
